# Correlation between the Gait Deviation Index and gross motor function (GMFCS level) in children with cerebral palsy

**DOI:** 10.1007/s11832-016-0738-4

**Published:** 2016-05-13

**Authors:** Merete A. Malt, Ånen Aarli, Bård Bogen, Jonas M. Fevang

**Affiliations:** Bergen Gait Laboratory, Haukeland University Hospital, Helse Bergen HF, Postboks 1400, 5021 Bergen, Norway; Department of Physiotherapy, Haukeland University Hospital, Helse Bergen HF, Postboks 1400, 5021 Bergen, Norway; Department of Paediatrics and Regional Centre of Habilitation and Rehabilitation in Western Norway, Haukeland University Hospital, Bergen, Norway; Department of Global Public Health Care and Primary Care, The University of Bergen, Postboks 7800, 5020 Bergen, Norway; Department of Orthopaedic Surgery, Haukeland University Hospital, Bergen, Norway

**Keywords:** Gait Deviation Index, Cerebral palsy, Gross Motor Function Classification System (GMFCS), Gait, Three-dimensional gait analysis

## Abstract

**Aim:**

The Gait Deviation Index (GDI) is a score derived from three-dimensional gait analysis (3DGA). The GDI provides a numerical value that expresses overall gait pathology (ranging from 0 to 100, where 100 indicates the absence of gait pathology). The aim of this study was to investigate the association between the GDI and different levels of gross motor function [defined as the Gross Motor Function Classification System (GMFCS)] and to explore if age, height, weight, gender and cerebral palsy (CP) subclass (bilateral and unilateral CP) exert any influence on the GDI in children with unilateral and bilateral spastic CP.

**Methods:**

We calculated the GDI of 109 children [73 % boys, mean age 9.7 years (standard deviation, SD 3.5)] with spastic CP, classified at GMFCS levels I, II and III. Twenty-three normally developing children were used as controls [61 % boys, mean age 9.9 years (SD 2.6)]. Multiple linear regression analysis was performed.

**Results:**

The mean GDI in the control group was 100 (SD 7.5). The mean GDI in the GMFCS level I group was 81 (SD 11), in the GMFCS level II group 71 (SD 11) and in the GMFCS level III group 60 (SD 9). Multiple linear regression analysis showed that gender, age and CP subclass had no significant correlation with the GDI, whereas height and weight had a slight impact.

**Conclusion:**

This study showed a strong correlation between the GDI and GMFCS levels. The present data indicate that calculation of the GDI is a useful tool to characterise walking difficulties in children with spastic CP.

## Introduction

Three-dimensional gait analysis (3DGA) is widely used to describe gait problems, as well as to plan and evaluate the treatment of children with cerebral palsy (CP). 3DGA provides a large amount of data in the form of graphs expressing joint motions (kinematics), as well as moment and power (kinetics) of the pelvis, hip, knee and ankle joints in three planes (sagittal, frontal and transverse) [1]. For clinical purposes, it is useful to summarise the results from 3DGA into a single, numerical measure that reflects the patient’s gait.

Several models have been designed to obtain a single measure of the quality of a gait pattern. Among these, the Gillette Gait Index (GGI) has been widely used. The GGI, however, has certain limitations. These include the component parameters used and difficulties in implementation to the control data. These limitations have been well documented in previous papers [2, 3].

Schwartz and Rozumalski published an article introducing the Gait Deviation Index (GDI) in 2008 [2]. The GDI is a score derived from 3DGA, which provides a numerical value that expresses overall gait pathology (range 0–100, where 100 and above indicates absence of gait pathology). Every 10-point decrease in the GDI corresponds to one standard deviation from the mean of healthy controls. They showed that the GDI corresponded with different levels of the Gillette Functional Assessment Questionnaire Walking Scale (FAQ). In other words, the GDI decreased with increased severity of CP [2].

Other studies demonstrated that reduced GDI correlated with increased disability, measured by the Gross Motor Function Measure (GMFM) and the Gross Motor Function Classification System (GMFCS) [4, 5]. The GDI has also showed good test–retest repeatability [4]. Weight and body mass index (BMI) do affect motor function in pre-school and school-aged children [6, 7] and could also affect the GDI. To our knowledge, there are no studies examining the influence of age, height, weight and gender on the GDI in children with CP.

The aim of this study was to investigate the association between the GDI and GMFCS level, as well as the possible influence of age, height, weight, gender and CP subclass on the GDI in children with unilateral and bilateral spastic CP.

## Methods

### Participants

**Fig. 1:**
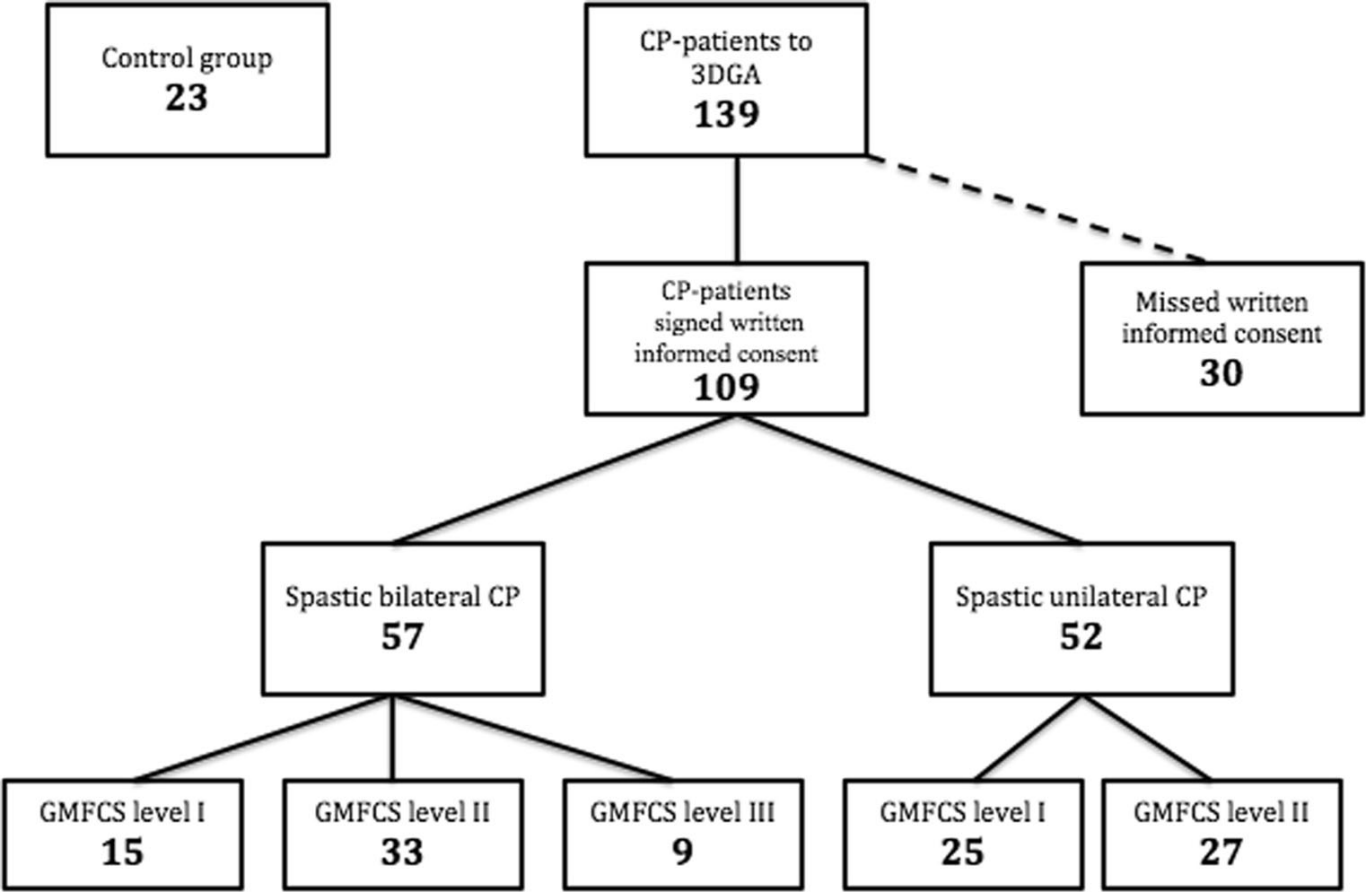
Participants in the present study. *CP* cerebral palsy; *3DGA* three-dimensional gait analysis; *GMFCS* Gross Motor Function Classification System

A request for permission to use data from previously completed gait analyses was sent to 139 children with spastic CP, who attended the gait laboratory at Haukeland University Hospital in the period from 01.03.2006 to 31.12.2013. A total of 109 patients were included in the study after written and informed consent (Fig. 1). The patients were classified according to GMFCS levels I (*n* = 40), II (*n* = 60) and III (*n* = 9). Fifty-seven children had bilateral CP, and 52 children had unilateral CP. For those children who had been to several gait analyses, only the first analysis was included in the study. A group of 23 typically developing children without any known motor dysfunction was used as controls.

Nine patients with spastic unilateral CP [8 boys/1 girl, mean age 7.5 years (standard deviation, SD 2.9)] and nine patients with spastic bilateral CP [4 boys/5 girls, mean age 8.3 years (SD 2.7)] had previously undergone orthopaedic surgery. The most common procedure was achilles tendon lengthening (14 patients). Three patients with spastic bilateral CP had undergone single-event multi-level surgery.

The study was approved by the Regional Ethics Committee for Medical Research in Norway (2012/1700).

### Data collection and analysis

3DGA data was collected using the Vicon Motion Systems with six infrared MX cameras and two AMTI OR6-7 force plates (Advanced Mechanical Technology, Inc., Watertown, MA, USA). Data was processed with Plug-in-Gait software for Workstation and NEXUS (Vicon Motion Systems, Oxford, UK). Reflective markers were placed according to the Plug-in-Gait model (Vicon Motion Systems, Oxford, UK). Participants walked barefoot at a self-selected speed along a 10-m walkway. All children walked independently and were allowed to use walking aids if needed. At least three trials with adequate force plate data were captured and processed for the right and left sides. If valid force plate data were not available, three trials that were considered to be representative for the walking pattern of the child were chosen. Three trials for every patient were exported from Polygon to Excel.

The GDI for the control group and CP patients were calculated as described by Schwartz and Rozumalski, by using the electronic addendum [2].

For patients with spastic bilateral CP, a mean GDI was calculated, which represented the average of the GDI for the right and left lower limbs. For the patients with spastic unilateral CP, only the GDI for the affected lower extremity was included.

The kinematic variables (maximum ankle dorsiflexion in stance, minimum knee flexion and minimum hip flexion in stance, and foot progression angle) were collected from the same trials from the 3DGA as used for the GDI calculation.

### GDI model

The GDI model captures kinematic components of the gait pattern and utilises pattern recognition to compare the average deviation from a control group. The GDI is developed using kinematic data from the pelvis, hip, knee, ankle and foot, from an average of several gait cycles. In the sagittal plane, the GDI incorporates kinematics from the pelvis, hip, knee and ankle joint. In the frontal plane, the GDI incorporates kinematics from the pelvis and hip joint. In addition, the kinematics from pelvis, hip and foot progression in the transversal plane are included in the GDI. In total, the GDI includes 459 kinematic data points (nine kinematic variables captured 51 times, i.e. every 2 % of the gait cycle). These 459 data points are compressed, and 15 parameters retrieved, to express an overall deviation, compared with 15 corresponding parameters from normal values in typically developing children [2].

### Statistical analysis

One-way analysis of variance (ANOVA) with the Bonferroni post hoc test was performed to explore the differences in the mean GDI between the control group (*n* = 23) and children with spastic CP GMFCS levels I (*n* = 40), II (*n* = 60) and III (*n* = 9).

To examine potential differences between the categorical variables, the Chi-square test was used.

To investigate the impact of age, height, weight and gender on the GDI, a multiple linear regression analysis was conducted (Table 3). The mean GDI was used as the dependent variable. GMFCS levels, height, weight, gender and CP subclass were used as independent variables.

Descriptive statistics, which compare the mean and the standard error of the mean, were used to explore differences among the most important kinematic variables (maximum ankle dorsiflexion in stance, minimum knee flexion and minimum hip flexion in stance, and foot progression angle) between the control group, spastic bilateral and unilateral CP, and GMFCS levels.

Statistical analyses were performed using SPSS for Windows statistical software (version 20.0). A significance level of *p* ≤ 0.05 and a confidence interval at 95 % were used for all statistical comparisons. Data are presented as means and SDs, unless otherwise indicated.

## Results

The patient characteristics, according to GMFCS level and CP subclass (unilateral or bilateral spastic CP), are presented in Table 1. There were no significant differences between the groups with respect to age, weight and gender. On average, the children in the control group were slightly taller than the children with CP, but this was not statistically significant.

**Table 1:** Characteristics of the patients and control group, Gross Motor Function Classification System (GMFCS) levels and subclass of cerebral palsy (CP)

	Control group, *n* = 23	Bilateral and unilateral CP			*p*-value	Bilateral CP, *n* = 57	Unilateral CP, *n* = 52
		GMFCS I, *n* = 42 (39 %)	GMFCS II, *n* = 58 (53 %)	GMFCS III, *n* = 9 (8 %)			
Bilateral/unilateral CP	0	15/25	33/27	9/0			
Gender, boys/girls	14/9	22/20	35/23	6/3	0.79^a^	32/25	31/21
Age, years (SD)	9.9 (2.6)	9.9 (3.5)	9.4 (3.5)	10.4 (10.4)	0.40^b^	9.7 (3.5)	9.6 (3.4)
Height, cm (SD)	141 (4.2)	135 (17.6)	130 (21.2)	130 (8.7)	0.07^b^	131 (18.3)	132 (20.2)
Weight, kg (SD)	35 (10)	36 (17)	31 (17)	31 (6.4)	0.88^b^	32 (14.2)	34 (18.6)
Earlier surgery	0	3	12	3		9	9

^a^Chi-square, cross-table diagnosis and gender

^b^Analysis of variance (ANOVA)

**Fig. 2:**
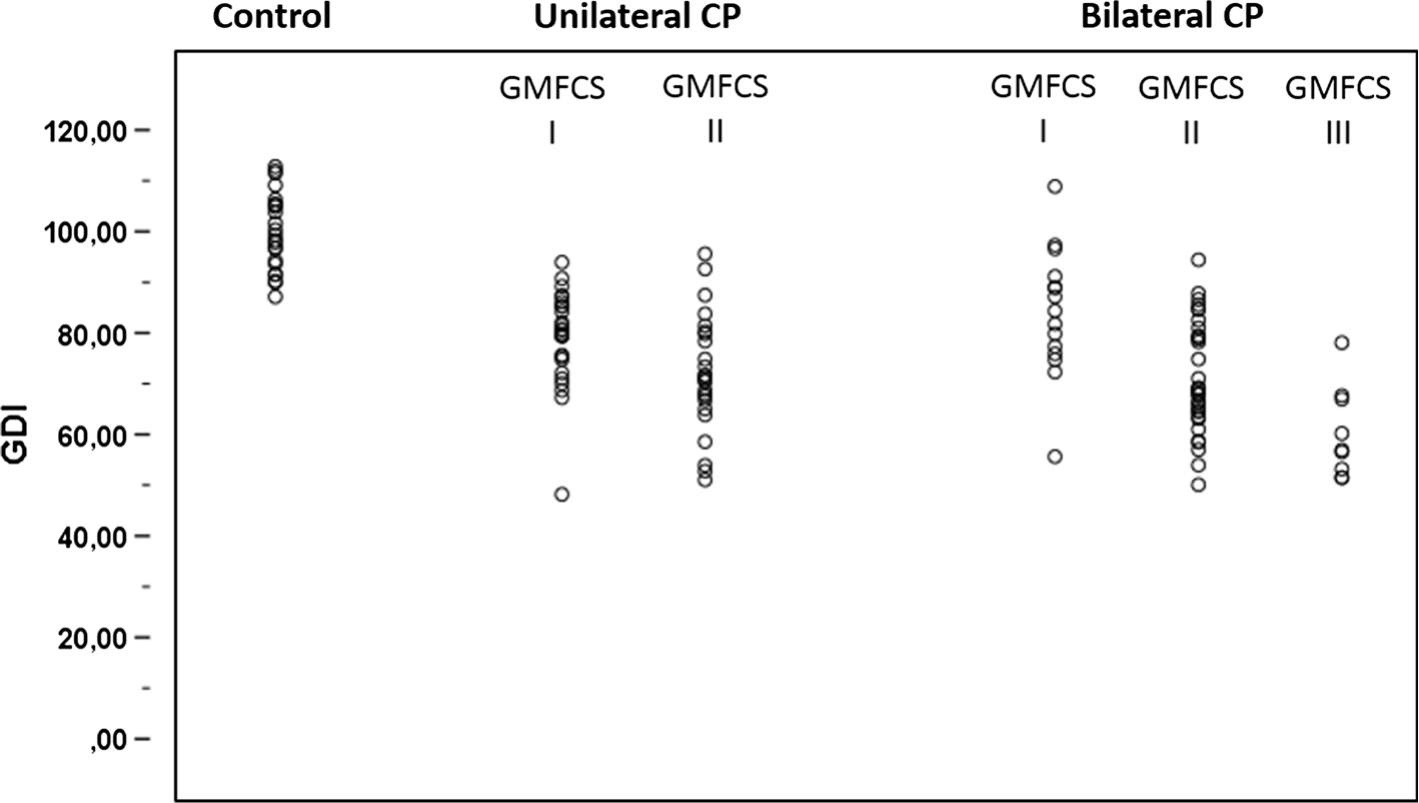
Scatter plot showing the distribution of the Gait Deviation Index (GDI) in the control group and patients with CP, GMFCS levels I–III

**Fig. 3:**
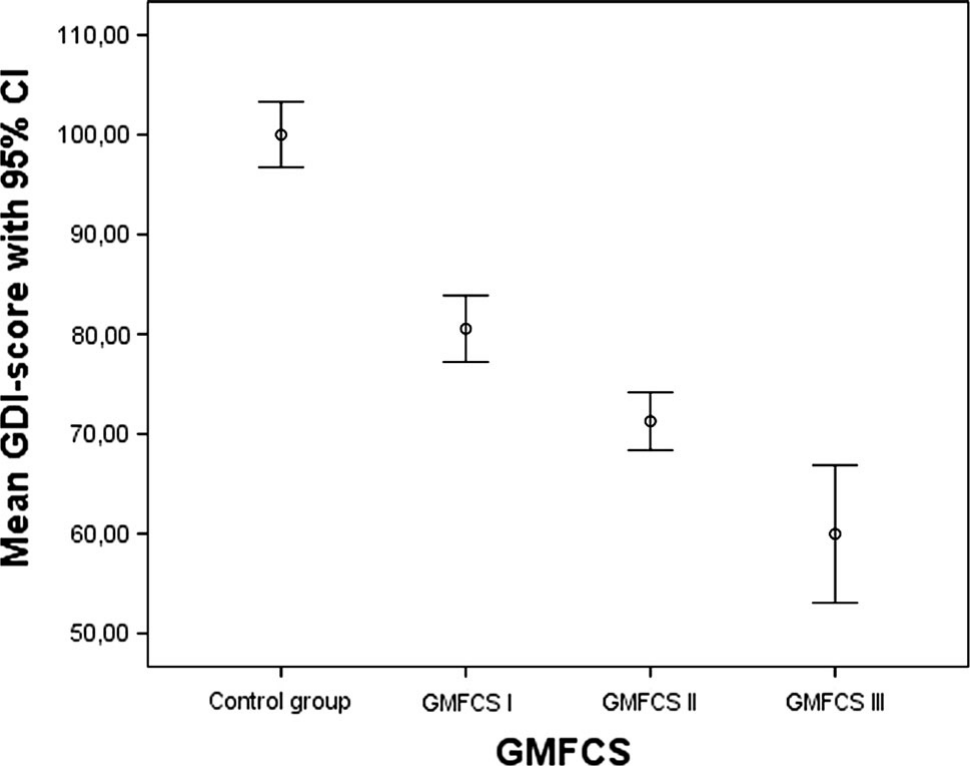
Mean GDI with 95 % confidence intervals in the control group and patients with CP, GMFCS levels I–III

Figure 2 shows the GDI for the control group and for the CP patients at GMFCS levels I, II and III. The figure demonstrates a considerable overlap between the study groups as, for example, two participants in the control group had a GDI below 90. The mean GDI with 95 % confidence interval (CI) for each study group is shown in Fig. 3. In the control group, the mean GDI was 100, in the GMFCS level I group 81, in the GMFCS level II group 7, and in the GMFCS level III group, the mean GDI was 60 (Fig. 3). In the subgroup analysis for patients with bilateral spastic CP, the mean GDI in the GMFCS level I group was 84, in the GMFCS level II group 71 and in the GMFCS level III group, the mean GDI was 60. For the patients with unilateral CP, the mean GDI on the affected lower extremity in the GMFCS level I group was 82 and in the GMFCS level II group it was 76. The difference between the control group and all adjacent GMFCS levels were statistically significant (*p* < 0.001). The results from the affected leg in patients with spastic unilateral CP did not differ significantly from the results from patients with spastic bilateral CP.

Subgroup analyses for patients with unilateral CP showed a significant difference between affected and contralateral limbs (*p* = 0.001), as expected. The mean GDI for the affected limbs was 75 (SD 10.8) and the mean GDI for the contralateral limbs was 82 (SD 10.9).

The influence of GMFCS level, age, height, weight, gender and CP subclass on the GDI is illustrated in Table 2. There was a strong correlation between the GDI and GMFCS levels (*p* < 0.001). Height and weight appeared to slightly influence the GDI. Taller participants tended to achieve higher GDI, whereas heavier patients had lower scores. In contrast, there was no significant correlation found between the GDI and gender, age and CP subclass. Boys had slightly higher GDI than girls (mean GDI 77 vs. 76, respectively).

**Table 2:** Possible predictors: Gait Deviation Index (GDI) level according to patient group (GMFCS level), age, height, weight, gender and bilateral CP/unilateral CP (multiple linear regression analysis)

Predictors	*R* ^2^, 0.309	*β*	95 % confidence interval		*p*-value
			Lower bound	Upper bound	
GMFCS level		−0.507	−13.61	−6.57	<0.001
Age (year)		−0.038	−0.800	0.527	0.68
Height (cm)		0.565	0.072	0.650	0.02
Weight (kg)		−0.459	−0.666	−0.021	0.04
Gender (female)		−0.111	−6.88	1.39	0.19
Bilateral/unilateral		0.055	−5.601	2.92	0.54

Table 3 shows the kinematic variables for the control group and for the patients with bilateral and unilateral CP at GMFCS levels I, II and III. The largest differences among the groups were seen in minimum knee flexion and minimum hip flexion in stance, where the patients with bilateral CP in GMFCS level III walked with increased hip and knee flexion. Patients with spastic bilateral CP in GMFCS levels II and III walked with internal foot progression.

**Table 3:** Clinical variables and mean GDI in patients with spastic unilateral and bilateral CP

	Control group, *n* = 23, mean GDI 100	Unilateral CP		Bilateral CP		
		GMFCS I, *n* = 25, mean GDI 82	GMFCS II, *n* = 27, mean GDI 76	GMFCS I, *n* = 15, mean GDI 84	GMFCS II, *n* = 33, mean GDI 71	GMFCS III, *n* = 9, mean GDI 60
Max. ankle dorsiflexion in stance	12.9° ± 0.6	8.8° ± 1.5	5.3° ± 3.0	7.7° ± 1.6	10.5° ± 1.6	5.8° ± 3.2
Min. knee flexion in stance	2.7° ± 0.9	0.7° ± 1.6	5.3° ± 2.1	2.2° ± 1.8	7.1° ± 1.9	18.9° ± 3.9
Min. hip flexion in stance	−12.2° ± 1	−6.8° ± 1.4	−1.1° ± 2.0	−7.0° ± 2.0	−3.3° ± 1.7	7.8° ± 3.2
Foot progression angle	−4.7° ± 1.5	2.0° ± 2.5	4.7° ± 3.4	−2.0° ± 2.6	9.1° ± 1.9	9.6° ± 5.3

## Discussion

This study confirms a strong association between the GDI and GMFCS levels. Parameters such as age, gender and CP subclass did not correlate significantly with the GDI, whereas height and weight had a slight impact.

The mean GDI of 100 (SD 7.6) for our controls and the lower GDI for the CP patients confirms previous publications on the GDI’s distributional properties [2, 5]. We found a strong association between GDI values and GMFCS levels I, II and III. This is in line with other publications [4, 5, 8]. The GDI appears to reflect different degrees of severity, although there is a considerable degree of overlap between the different GMFCS levels in the distribution of the GDI. In the healthy control group, two patients had mean a GDI between 80 and 90. More than 50 % of children with CP at GMFCS level I achieved a mean GDI above 80 points, which is within 2 SD of the control group. At GMFCS level II, 24 % of patients had a mean GDI above 80, but at GMFCS level III, no patients achieved a mean GDI above 80. These results reflect individual variation within each GMFCS level. Some individuals with spastic CP at GMFCS level I did, in fact, demonstrate an almost normal gait pattern based on the GDI.

For children with unilateral spastic CP, the GDI on the affected side were significantly lower than on the contralateral side, as expected. The GDI from the contralateral unaffected side were lower than the controls, which is in agreement with other studies [2, 9].

We also found that the covariates age, gender and CP subclass were not significantly correlated to the GDI, whereas height and weight had a slight impact.

The GDI was positively correlated with height and negatively correlated with weight. To our knowledge, this has not been reported in other studies. It seems conceivable that increased height and leg length is positively correlated with walking speed, and there is evidence that kinematics and kinetics are speed-dependent [10–12]. The negative influence of increased weight could be explained by overweight, complicating gait pattern in some individuals.

In this study, age or gender did not correlate with the GDI. As far as we know, this has not been reported earlier. However, Rutz et al. showed that age and gender did not significantly affect the outcome after surgery on Gait Profile Score (GPS) [8].

Children with CP reach a plateau in their motor development at around 7 years of age [13, 14]. The patients included in our study had a mean age of 9.7 years (SD 3.4) and would, thus, be expected to have reached a stable level of gross motor function. If confirmed by other studies, the notion that the GDI is independent of age may be helpful in the long-term follow-up of patients.

The patient cohort was not selected and showed a clear predominance of males (77 of 132). This is in line with epidemiological studies that demonstrate an unexplained male preponderance in all CP subclasses except ataxia [15].

The kinematic variables presented in this study showed more deviation in patients with spastic bilateral CP in GMFCS levels II and III, especially in knee and hip flexion (crouch gait). As expected, these patients also had lower GDI. This indicates that the GDI corresponds with reduced walking ability and increased GMFCS level.

Although the gait pattern is obviously quite variable among children with CP [16, 17], this study does not demonstrate any significant influence on the GDI by CP subclass itself, only by GMFCS level.

## Conclusion

In conclusion, our study indicates that calculation of the Gait Deviation Index (GDI) is a useful tool to characterise walking difficulties in children with spastic cerebral palsy (CP). The GDI is strongly associated with the Gross Motor Function Classification System (GMFCS) level. There was no significant influence of age, gender and CP subclass (bilateral and unilateral CP) on the GDI in our patient group, whereas height and weight had a slight impact.
